# Topical tacrolimus in anterior segment inflammatory disorders

**DOI:** 10.1186/s40662-017-0072-z

**Published:** 2017-03-09

**Authors:** Samir S. Shoughy

**Affiliations:** The Eye Center and the Eye Foundation for Research in Ophthalmology, PO Box 55307, Riyadh, 11534 Saudi Arabia

**Keywords:** Tacrolimus, Topical, Ocular, Immune-mediated, T cells

## Abstract

Immune mediated inflammatory anterior segment diseases are variable and their management requires intense immunosuppression. Treatment with topical steroids is associated with serious ocular side effects. In order to overcome the potentially blinding complications of topical steroids, immunomodulatory drugs are being used more frequently. Tacrolimus is a calcineurin inhibitor that induces suppression of T lymphocytes activity and reduction of ocular inflammation. Tacrolimus was recently investigated for application in various anterior segment inflammatory disorders. In this review, we will discuss the therapeutic application of topical tacrolimus as a steroid-sparing agent in treating T cell mediated anterior segment inflammation.

## Background

Immune mediated inflammatory anterior segment diseases are common and their management requires intense immunosuppression. Topical and/or systemic steroids are the mainstay to control inflammation in these diseases. However, prolonged use of steroids may lead to development of serious and sight-threatening complications including cataract, glaucoma and increased susceptibility to infection. In order to overcome the potentially blinding complications of topical steroids, immunomodulatory drugs are being used more frequently.

Tacrolimus was discovered in a soil sample taken from the foot of Mount Tsukuba in Ibaraki in 1984 [1]. Tacrolimus, also known as (FK506), is a macrolide produced from the fermentation broth of a Japanese soil sample that contained the bacteria *Streptomyces tsukubaensis* [2]. The generic name is a neologism composed of “Tsukuba macrolide immunosuppressive” [3]. It was among the first macrolide immunosuppressants discovered and was found to have potent in vitro immunosuppressive effects [1]. The efficacy of tacrolimus as an immunosuppressive agent was reported in 1986 at the 11^th^ World Congress of the Transplantation Society in Helsinki, Finland, by researchers from Chiba University, Japan. Within 5 years of its discovery, clinical trials were initiated for tacrolimus use in transplant rejection to reduce immune system activity and to lower the risk of organ rejection following transplantation [1]. Later on, topical tacrolimus was approved for the treatment of atopic dermatitis in Japan in 1990, US in 2000 and in Europe in 2001 [4].

Tacrolimus binds to FK506-binding proteins within T lymphocytes and inhibits calcineurin activity. Calcineurin inhibition suppresses dephosphorylation of the nuclear factor of activated T cells and its transfer into the nucleus, which results in the suppressed formation of cytokines by T lymphocytes [5, 6]. Inhibition of T lymphocytes may therefore lead to the inhibition of release of inflammatory cytokines and decreased stimulation of other inflammatory cells [6].

Based on the immunosuppressive properties of tacrolimus, several clinical trials were conducted to assess its efficacy for ophthalmic use. Different forms and concentrations of tacrolimus have been assessed in the treatment of anterior segment inflammatory disorders (Tables 1 and 2). Dermatological preparations (Protopic ointment, Astellas Phama, Tokyo, Japan) were FDA-approved for the treatment of atopic dermatitis. The off-label use in a spectrum of variable ophthalmic conditions has been reported as safe and effective. The selection of concentration, form and frequency depends on the disease entity and its severity [7]. The majority of the previous studies have focused on allergic eye disease [5]. In the following review, we will discuss the applications of topical tacrolimus in various T cell mediated ocular diseases.

**Table 1:** Topical tacrolimus in allergic eye diseases

Disease	Reference/Authors	No. of eyes (patients)	Tacrolimus form	Frequency	Study design
AKC, VKC	11/Ohashi et al.	56 (28)	Suspension 0.1%	2 times	Prospective
AKC	12/Al-Amri et al.	22 (11)	Ointment 0.1%	Variable	Prospective
AKC, VKC	13/Fukushima et al.	2872 (1436)	Suspension 0.1%	2 times	Prospective
VKC	14/Vichyanond et al.	20 (10)	Ointment 0.1%	1–2 times	Prospective
AKC, VKC	15/Miyazaki et al.	1582 (791)	Suspension 0.1%	2 times	Prospective
VKC	5/Shoughy et al.	124 (62)	Solution 0.01%	2 times	Retrospective
AKC, VKC	16/Miyazaki et al.	12 (6)	Ointment 0.02%	1–4 times	Retrospective
VKC	18/Kheirkhah et al.	20 (10)	Solution 0.005%	4 times	Prospective

*VKC* = Vernal keratoconjunctivitis; *AKC* = Atopic keratoconjunctivitis

**Table 2:** Topical Tacrolimus in anterior segment disorders

Disease	Reference/Authors	No. of eyes (patients)	Tacrolimus form	Frequency	Study design
CAU	20/Taddio et al.	6 (3)	Solution 0.1%	3 times	Case series
Scleritis	16/Miyazaki et al.	2 (2)	Ointment 0.02%	1–4 times	Retrospective
Scleritis	31/Lee et al.	4 (4)	Ointment 0.02%	1–4 times	Retrospective
GVHD	32/Jung et al.	24 (13)	Ointment 0.02%	1–2 times	Retrospective
GVHD	33/Tam et al.	2 (1)	Ointment 0.03%	2 times	Case report
GVHD	34/Ryu et al.	14 (7)	Ointment 0.02%	2 times	Prospective
GVHD	35/Abud et al.	48 (24)	Suspension 0.05%	2 times	Prospective
OCP	39/Hall et al.	2 (1)	Ointment 0.03%	once daily	Case report
OCP	40/Michel et al.	2 (1)	Ointment 0.03%	once daily	Case report
OCP	31/Lee et al.	2 (1)	Ointment 0.02%	1–3 times	Retrospective
SJS	31/Lee et al.	11 (5)	Ointment 0.02%	1–3 times	Retrospective
SLK	44/Kymionis et al.	4 (2)	Ointment0.03%	2 times	Case report
AK	47/Ghanem et al.	10 (7)	Suspension 0.03%	2 times	Prospective
AK	48/Levinger et al.	11 (11)	Ointment 0.03%	2 times	Prospective
Dry Eye	50/Moscovici et al.	48 (42)	Suspension 0.03%	2 times	Prospective
PKC	51/Kymionis et al.	2 (2)	Ointment 0.03%	2 times	Case report
PKP	59/Dhaliwal et al.	4 (4)	Ointment 0.03%	2 times	Case series
PKP	60/Magalhaes et al.	36 n	Suspension 0.03%	2 times	Retrospective
PKP	61/Reinhard et al.	20 20)	Solution 0.06%	3 times	Prospective

*CAU* = chronic anterior uveitis; *GVHD* = Graft Versus Host Disease; *OCP* = Ocular Cicatricial Pemphigoid; *SJS* = Stevens-Johnson syndrome; *SLK* = Superior limbic keratoconjunctivitis; *AK* = Adenoviral Keratitis; *PKC* = Phlyctenular keratoconjunctivitis; *PKP* = Penetrating keratoplasty

## Review

### Topical tacrolimus in allergic eye disease

Th2 lymphocytes play a pivotal role in the pathogenesis of vernal keratoconjunctivitis (VKC). The levels of Th2-derived cytokines including mRNA for IL-3, IL-4, IL-5 and IL-13 are increased in patients with VKC [7]. Furthermore, Th2 lymphocytes induce IgE production by stimulation of B lymphocytes, and lead to activation of mast cells, eosinophils and neutrophils [7]. In atopic keratoconjunctivitis (AKC) the cell-mediated response is different from those in VKC. In AKC, there is expression of both Th1 and Th2 cytokines in the inflamed conjunctiva with potential involvement of Th1-mediated mechanisms [6]. Inhibition of T lymphocytes by tacrolimus may therefore, lead to inhibition of release of inflammatory cytokines and decreased stimulation of other inflammatory cells. In addition, the immune-suppressive effects of tacrolimus are not limited to T lymphocytes, but it may also act on B cells, neutrophils and mast cells leading to improvement of symptoms and signs of VKC [8–10].

Different forms and concentrations of tacrolimus have been assessed in the treatment of allergic eye diseases including refractory VKC and AKC (Table 1). The main concentration of topical tacrolimus that was investigated in the majority of the clinical trials was 0.1% [11–15]. Some other studies evaluated lower concentrations of tacrolimus including 0.005, 0.01, 0.02 and 0.03% [5, 16–18]. These studies showed that even with low concentrations of tacrolimus, the topical eye drop was a safe and effective treatment modality for patients with VKC refractory to conventional medications including topical steroids. There was dramatic improvement of symptoms of itching, redness, photophobia, ocular discomfort, foreign body sensation and tearing. Similarly, there was improvement of signs of conjunctival hyperemia, conjunctiva papillae, limbal infiltration, Trantas’ dots, and superficial punctate keratopathy. In addition, many patients were well controlled on topical tacrolimus alone without addition of other medications. However, it was noted that long-term use of the medication was necessary to control the disease [18]. Any attempt to discontinue topical tacrolimus during active disease is associated with immediate recurrence of symptoms [5].

### Topical tacrolimus in anterior uveitis

Uveitis is a sight threatening inflammatory disorder that affects all ages and remains a significant cause of visual loss [6, 19]. Uveitis may be idiopathic or associated with underlying systemic disease. These systemic illnesses may be infectious or driven by autoimmune mechanisms [6, 20]. Several forms of uveitis are believed to be mediated by T cells [6].

Since tacrolimus inhibits T-cell proliferation and suppresses release of inflammatory cytokines, it can theoretically be used to reduce inflammatory activity in uveitis patients [6]. Several studies were conducted to assess the efficacy of tacrolimus for treatment of immune-mediated uveitis. The topical form of tacrolimus was initially evaluated in animals [21, 22]. It was found to be effective in inhibiting endotoxin-induced uveitis and autoimmune uveitis in experimental uveitis models [21, 22]. However, tacrolimus has difficulty penetrating the corneal epithelium and accumulates in the corneal stroma due to its poor water solubility and relatively high molecular weight [23]. There have been several trials aimed at improving corneal penetration and prolonging precorneal retention time. Nanoscale drug delivery systems, such as nanoparticles, cubosomes, nanoemulsions, and liposomes, have shown to improve corneal penetration and increase retention time [19, 24]. Intravitreal injection of tacrolimus was similarly found to be highly effective in suppressing the ongoing process of endotoxin-induced uveitis in animal studies. This has led to the assumption that tacrolimus may be useful in the management of patients with uveitis [25].

Based on its success in the treatment of uveitis in animal models, several trials were conducted to evaluate the efficacy of systemic therapy with tacrolimus in refractory uveitis [26–28]. Studies that evaluated topical tacrolimus in humans are scarce. Taddio et al. demonstrated that topical tacrolimus 0.1% was effective in controlling intraocular inflammation in 3 children with anterior uveitis with coexistent VKC [20]. The potential role of topical tacrolimus in treating patients with uveitis is yet to be determined. Further studies are needed to detect the optimum concentration and formula of topical tacrolimus.

### Topical tacrolimus in scleritis

Normally, the human sclera contains few or no macrophages, Langerhans’ cells, neutrophils, or lymphocytes. After scleral inflammation, there is a marked increase in T-helper lymphocytes with a high T-helper to T-suppressor ratio. These findings suggest that T lymphocytes may play a role in some forms of scleritis [29]. Tacrolimus, being a T lymphocyte inhibitor, may therefore ameliorate inflammation in certain forms of scleritis. The use of tacrolimus in the treatment of scleritis is not well-documented. Young et al. reported success of systemic tacrolimus in treating resistant surgically induced necrotizing scleritis [30]. Miyazaki et al. found that topical tacrolimus ointment had an additive therapeutic effect to topical steroid and helped to significantly reduce the scleral inflammation in 2 patients with sclerokeratitis [16]. In a study by Lee et al., it was similarly found that the supplemental use of topical tacrolimus ointment on the previous systemic and topical steroid therapy, without adding systemic immunomodulatory therapy, could calm active inflammation and help to taper oral and topical steroid within 3 months in patients with scleritis [31].

Therefore, topical tacrolimus could be used safely and effectively to treat certain forms of scleritis particularly steroid responders and as an adjunctive to systemic medications. It may also allow the use of a weaker topical steroid to avoid elevation of IOP or cataract development. However, it should be emphasized that extreme effort should be taken to diagnose and treat underlying systemic diseases as tacrolimus may ameliorate sclera inflammation only leaving the associated systemic illness poorly managed.

### Topical tacrolimus in GVHD

Chronic ocular graft versus host disease (GVHD) occurs in about 50% of transplant recipients after hematopoietic stem cell transplantation [32]. The clinical spectrum of chronic ocular GVHD includes dry eyes and chronic conjunctival inflammation such as pseudomembranous and cicatricial conjunctivitis and blepharitis. Although the pathogenesis of ocular GVHD is still unclear, the inflammatory processes of the lacrimal gland and ocular surface seem to play important roles [32]. Both allo- and auto-reactive T cells are thought to be involved. Tacrolimus is a calcineurin inhibitor and blocks T cell activation by inhibiting transcription initiation of specific genes [32]. Therefore, tacrolimus may reduce severe ocular surface inflammation that results from cell mediated immune reaction. Tacrolimus is hydrophobic and has a high molecular weight, which could allow it to permeate the conjunctiva more than the cornea [22]. The conjunctiva is up to 20 times more permeable to lipophilic and high-molecular-weight drugs than is the cornea. This could explain the higher efficacy of tacrolimus in patients with ocular GVHD with severe conjunctival inflammation [22]. Hence, topical tacrolimus could be used as an adjunctive therapy in patients with GVHD to minimize the duration and dose of topical steroid.

The efficacy of topical tacrolimus for treatment of ocular GVHD has been reported in few previous studies. Jung et al. studied 24 eyes of 13 patients with GVHD. Patients were treated with tacrolimus ointment for up to 20 months [32]. The ocular surface inflammatory score decreased and the need for steroid treatment also decreased after initiating tacrolimus treatment. Tam and associates demonstrated the efficacy of a 1-month use of topical 0.03% tacrolimus ointment in controlling initial inflammation in a single patient with chronic ocular GVHD [33]. Ryu and associates reported the therapeutic effect of 0.03% tacrolimus ointment in 14 eyes of 7 patients with refractory anterior segment inflammatory disease associated with GVHD [34]. Recently, Abud et al. found that tacrolimus is a safe and effective therapeutic agent for the treatment of ocular manifestations of GVHD without the known ocular hypertensive effects of topical steroids [35].

### Topical tacrolimus in cicatrizing conjunctivitis

Ocular cicatricial pemphigoid (OCP) is a progressive cicatrizing conjunctivitis that may lead to fornix foreshortening, symblepharon formation, trichiasis, dry eye syndrome, corneal scars, ankyloblepharon, and blindness [36]. Evidence that cicatricial pemphigoid is an autoimmune disease is considerable [37]. By elaborating cytokines that promote fibroplasia, the T cells in OCP may be effector cells along with other types of inflammatory cells in bringing about the scarring of the conjunctiva. Furthermore, T cells may be responsible for inducing local B lymphocytes to produce autoantibodies to the epithelial basement membrane [38]. Experience in treating ocular disease with topical tacrolimus has been reported. Hall et al. and Michel et al. reported successful use of topical tacrolimus in the treatment of patients with OCP [39–41]. Lee et al. evaluated the therapeutic efficacy of topical tacrolimus in one patient with OCP and 6 cases of Stevens-Johnson syndrome (SJS). They found that despite the incomplete effect of topical tacrolimus, it contributed to the improvement of epithelial regeneration, ocular pain, and the progression of symblepharon or corneal neovascularization while tapering off topical steroids [31].

### Topical tacrolimus in other ocular surface diseases

Superior limbic keratoconjunctivitis (SLK) is a disease characterized by inflammation of the upper palpebral and superior bulbar conjunctiva [42]. Conjunctival specimens from patients with SLK have been shown to have an alteration in T-cell functions [42, 43].

Given the success of tacrolimus in several T-cell mediated ocular pathologies, it follows that topical tacrolimus may have utility in the treatment of SLK. Kymionis et al. reported success of topical treatment in improving ocular symptoms and controlling surface inflammation with resolution of superior conjunctiva hyperemia, papillary reaction and punctate keratopathy in patients with SLK [44].

The subepithelial infiltrates in patients with adenoviral keratoconjunctivitis represent a cellular immune reaction against viral antigens deposited in the corneal stroma under the Bowman’s membrane and can persist for weeks to years [45]. Immune response against viral replication in subepithelial keratocytes is responsible for the subepithelial infiltrates [45]. Histologically, these infiltrates are composed of lymphocytes, histiocytes, and antigen-presenting Langerhans cells [46]. Therefore, topical tacrolimus may be considered an effective treatment regimen. Topical tacrolimus 0.03% was found to be an effective corticosteroid-sparing agent for the treatment of patients with symptomatic corneal subepithelial infiltrates secondary to adenoviral keratoconjunctivitis [47, 48].

The evidence from previous studies suggest that the ocular inflammation in dry eye disease is mediated by T lymphocytes. Accordingly, clinical symptoms of dry eye may be dependent on T-cell activation and subsequent autoimmune inflammation. Tacrolimus inhibits T lymphocyte and suppresses the immune response by inhibiting the release of other inflammatory cytokines as well (e.g., IL-3, IL-4, IL-5, IL-8, interferon-gamma, and TNF-alpha). The reduction in inflammation through inhibition of T-cell activation and down-regulation of inflammatory cytokines in the conjunctiva and lacrimal gland may therefore enhance tear production [49]. In a prospective double-blind randomized study, topical tacrolimus was found to be effective in improving tear stability and ocular surface status in patients with dry eye syndrome [50]. Topical tacrolimus was also considered in treating severe refractory phlyctenular conjunctivitis and contact lens related papillary conjunctivitis [51, 52].

### Topical tacrolimus following corneal transplantation

Corneal transplantation is a commonly performed ophthalmic procedure. Graft rejection still represents a major threat for the graft. Low-risk grafts have a good prognosis, with a rejection rate of approximately 13.5% within 2 years. Topical steroid therapy usually ensures survival of low-risk corneal grafts [53]. On the other hand, the reported failure in high-risk grafts rates were between 60 and 90% depending on the criteria used to define high risk. Patients under high risk have been defined as having at least two quadrants of stromal vascularization and/or a history of previous graft rejection. Other risk factors include herpes simplex virus keratitis, chemical burns, large grafts, glaucoma, peripheral anterior synechiae, and younger age of the recipient [53]. Corneal graft rejection is a T cell-mediated immune response. Therefore, immunosuppressive drugs with T cell inhibitory action such as tacrolimus could be considered in the prevention and management of corneal graft rejection [54]. Several studies have shown enhanced graft survival in animal models of high-risk grafts using topical tacrolimus [55–58].

In humans, topical tacrolimus was evaluated for its efficacy following keratoplasty especially for high- risk corneal grafts. It demonstrated efficacy in preventing new episodes of graft rejection [59] and irreversible rejection [60]. Topical tacrolimus was also evaluated for normal-risk keratoplasty and was found to provide effective immunoprophylaxis [61]. Systemic use of tacrolimus was also evaluated. It may be a safe and effective therapeutic modality in reducing rejection and prolonging graft survival in patients with high-risk keratoplasty [53, 62, 63].

## Side effects of topical tacrolimus

Topical tacrolimus is generally well tolerated. The most common side effect is mild and transient ocular irritation [11]. Burning sensation may occur upon drop instillation but usually does not necessitate discontinuation of treatment. As an immunosuppressant, topical tacrolimus use may be associated with increased risk of corneal infections with prolonged use [11]. The incidence of corneal infection in a large cohort of patients treated with topical tacrolimus was 0.35% [13]. Corneal infections may be in the form of bacterial keratitis or herpetic corneal ulcer [13]. Accordingly, close monitoring of patients on prolonged topical tacrolimus therapy is mandatory. This is particularly necessary in patients with atopic dermatitis as they are more likely susceptible to herpes simplex virus infection [11, 13]. Atopic dermatitis patients treated with tacrolimus skin ointment may also have an increased risk of T-cell lymphoma [64]. Young patients with a higher body surface area per weight and patients with skin abnormalities of the epidermis may have considerable percutaneous absorption of tacrolimus ointment. The blood concentrations may be of sufficient level to induce immunosuppression [64]. Therefore, caution may be required especially for young patients with concomitant use of tacrolimus ointment. In such patients, monitoring the blood level of tacrolimus may be required [64, 65]. Further studies are needed to detect if there is a causal relationship between topical application of tacrolimus and the development of lymphoma. Such studies should address the possible risk factors including patient age, dose of tacrolimus, blood level of tacrolimus following topical use, and underlying systemic condition. In a study by Ebihara et al., it was demonstrated that based on the blood concentration profile of tacrolimus, systemic exposure was minimal and transient after topical application of the 0.1% ocular preparation [65]. In fact, the theoretical risk of systemic adverse effects due to exposure to topical tacrolimus is very low [65].

## Conclusion

In conclusion, topical tacrolimus is a promising drug for treating T cell mediated anterior segment ocular diseases. More studies are needed to define the optimal formula and concentration to achieve adequate control of inflammation.
